# Microwave System: A Novel Treatment for Localized Adiposity Reduction in a Latin American Population

**DOI:** 10.1155/2023/9998499

**Published:** 2023-07-11

**Authors:** Aura Ibeth Ruiz-Rosas, Nelly Patricia Muño-Velasco, Dayana Sofia Rengifo-Bolaños, Tatiana Carolina Reyes-Vivas, Paula Lozano-Bitar, Irene Fusco, Paola Andrea Russy-Buitrago

**Affiliations:** ^1^Aesthetic Physician, Aesthetic Dermatology Service, Laser Medical Care Clinic, Bogotá, Colombia; ^2^Professor of Dermatology, National University of Colombia, Bogotá, Colombia; ^3^Dermatology Resident, National University of Colombia, Bogotá, Colombia; ^4^Specialist and Master in Clinical Epidemiology, University Foundation of Health Sciences, Bogotá, Colombia; ^5^El.En. Group, 50041 Calenzano, Florence, Italy; ^6^Master in Clinical Epidemiology, Resident Dermatology, National University of Colombia, Bogotá, Colombia

## Abstract

**Background:**

The microwave body remodeling system is indicated for people who want to improve their physical appearance as it can penetrate deep tissues, causing thermic stress on adipocytes to produce adipolysis while in superficial tissues, it dissolves fibrous tracts and stimulates new collagen.

**Objectives:**

The aim of this study was to assess the localized adiposity reduction in Latin American patients using a microwave system.

**Methods:**

A total of 35 patients with a mean age of 47.5 (±9.0) years received body remodeling treatment, using the microwave system between the years 2019–2022 in a Bogota, Colombia reference center. Data descriptive analysis was made as well as single-factor repeated measures ANOVA to show pre- and post-treatment difference, and mixed ANOVA for body mass index (BMI) subgroup analysis was performed.

**Results:**

In all patients examined, statistical significant differences were found in pre- and post-treatment skinfold test for each body area: superior abdomen (*F*(1,27) = 63.13; *p*=0.001), iliac crest (*F*(1, 23) = 114.33; *p* < 0.001), posterior waist (*F*(1, 20) = 27.36; *p* < 0.001), trochanter (*F*(1, 17) = 26.94; *p* < 0.001), among others.

**Conclusions:**

According to the study's findings, this microwave system is an innovative and effective technique for body remodeling and cellulite and localized fat reduction.

## 1. Introduction

**Figure 1:**
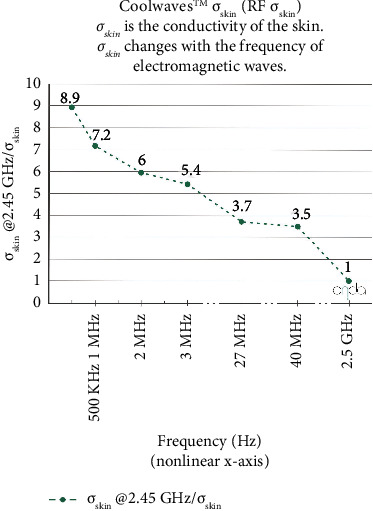
Graphical representation of study device conductivity. Courtesy of DEKA M.E.L.A company (from white paper, “how the new onda system works: the coolwaves^tm^ effect”, https://www.lynton.co.uk/wpcontent/uploads/2020/04/Onda_DekaWP_July2018.eng_.rev1_.1.pdf).

The microwave body remodeling system constitutes a novel noninvasive technology for subcutaneous adipose tissue reduction [1, 2]. Designed for deep tissue heating, it provides treatment of localized adiposities, edematous fibro sclerotic panniculopathy (EFP), and skin laxity, among other body remodeling applications in the fields of dermatology, plastic surgery, and aesthetic medicine [1, 3]. A frequency of 2.45 GHz in a controlled microwave emission interacts with biomolecules and generates controlled, localized heat where 80% of the transferred energy is absorbed in a selective manner by adipocytes in the deepest layer of the skin through a biophysical process called “dielectric heating” [2, 4, 5]. The immediate consequence is a disarray of the adipocyte's cytoplasm due to a metabolic increase with an outflow of the fatty content through the blebbing effect [2] producing physiologic macrophage activation that takes up this fat and eliminates it via lymphatic drainage, at the same time producing collagen solubilization, fibroblasts activation, and fibrous tissue remodeling, reducing adipose tissue, improving cellular metabolism, local circulation, and skin quality [2]. This way, the system induces thermic modifications in subcutaneous adipose tissue, preferably without affecting the dermo-epidermic layer, unlike other systems such as radiofrequency (RF) [1]. In fact, the selective microwaves (called Coolwaves) of the Onda device (which works at 2.45 GHz) thanks to this high frequency, make the skin tissue almost “transparent” to the passage of energy which is almost totally conducted over the subdermal fat layer specifically. This makes the superficial layers of the dermis to be preserved from unwanted heating to stay cool. 80% of this selective microwave energy targets the fat cells, and 20% of energy is absorbed by the epidermal and dermal layers (this is in any case counterbalanced by the cooling system integrated into the handpiece that limits the effects of such heat and protects the epidermis). The situation is quite different with RF handpieces with a high risk of skin damage. Moreover, as the RF energy remains close to the surface, it fails to reach the hypodermis where the fat cells are located, and whose membranes must be broken for the treatment to be effective. For these microwaves, the conductivity of the outermost layers of skin is at least 3.5 times higher than that of the commonly used RF irradiation systems in aesthetic medicine (Figure 1). That means that most of the RF energy gets stuck in the epidermis and dermis, heating them up to such an extent that there is a risk of tissue damage.

The main application of this system is the management of EFP, commonly known as cellulite. This condition affects between 80 and 90% of the female postpubertal population [1], and it is manifested as an alteration in the skin surface consisting of depression and elevation that give an irregular appearance known as “orange skin”. Typically, it is found on the thighs, buttocks, arms, and abdomen causing aesthetic unconformity [1]. It is caused by soft tissue fibrosis and sclerosis induced by genetic and hormonal factors, alcohol ingestion, contraceptive use, and microcirculatory venous lymphatic dysfunction among others. These factors are persistent despite a healthy diet and exercise [4]. The predominance in females is due to the structural and anatomical characteristics of the subcutaneous septum, where the fibers are oriented perpendicular to the skin surface generating a thick division between fat lobules, unlike males where the septum has an interlaced pattern; additionally, estrogen favors fat accumulation, especially in the lower half of the body, and gives the characteristic female “pear-shaped” morphology [6].

Obesity is related to generalized adiposity, defined by the World Health Organization (WHO) as a body mass index superior to 30 kg/m^2^, and its management must be carried out with additional measures by a multidisciplinary team [7, 8]. Although cellulite can affect individuals with normal weight, it is worsened by overweight and obesity. In Colombia, the prevalence for adults in the year 2020 was 57.5%, of which 36.2% corresponded to being overweight and 21.3% to obesity, and it is two times more common in females (65.4%) than in males (34.6%) [9, 10].

In the last 30 years, minimally invasive technologies based on energy such as RF, low-frequency ultrasound, cryolipolysis, and combined infrared light therapy are on the rise; among them, the introduction of the microwave system for body remodeling, with reports of in vitro studies, animal models, and case series that show promising results such as those by Di Pietro et al. [1], which detailed the improvement after four 30 minute sessions in the cellulite severity scale and the findings by Bonan et al. [4], showed a median reduction in abdominal circumference of 3.9 cm eight weeks post treatment.

These published studies demonstrate the beneficial effect of microwave at 2.45 GHz on the reduction of subcutaneous fat and for skin laxity and cellulite improving in Caucasian populations. In this study, we present for the first-time clinical data on the effect of the Onda (DEKA M.E.L.A, Florence, Italy) for localized adiposity reduction in a Latin American population.

## 2. Materials and Methods

### 2.1. Study Design

Cross-sectional analytical study data were collected from a medical aesthetic center in Bogotá between January 2019 and April 2022.

### 2.2. Population

Adults older than 18 years that consulted the aesthetic medicine office voluntarily for localized adiposity reduction using microwaves for a minimum of three sessions were included consecutively. Patients with pregnancy less than four months postpartum, incomplete medical record, no follow-up after the third session, any medical contraindications to the procedure, and a lack of desire to participate in the study were excluded.

### 2.3. Instrument and Data Recollection

For data recollection, an instrument validated by the aesthetic medical center was used, and it included all the evaluated variables. Patient personal data were collected by trained nurses, and the clinical evaluation and anthropometric data were collected by the treating physician (the same in all cases).

### 2.4. Variables

The variables of interest were classified as sociodemographic, clinical, and device-specific. Sociodemographic variables were age (years), occupation, socioeconomic stratum, and educational level; clinical variables included past medical history of aesthetic surgery in the body, area of interest, gynecological history such as menopause or use of contraception, and anthropometric measurements were taken before and after treatment and included BMI (weight (kg)/height (m^2^) taken with the patient in underwear with a weight and height meter; abdominal perimeter (cm) taken standing with a flexible measuring tape midpoint between the inferior costal margin (inferior border of the 10th rib) and the iliac crest (anterosuperior iliac spine) between the end of expiration and the beginning of the inspiration of the respiratory cycle in accordance to the international directives [10]; skinfold measurement with a body fat caliper (mm) was taken by holding it firmly between the thumb and index fingers; evaluated skinfolds included tricipital, subscapular, abdominal, and iliac crest, measured using the recommendations in the protocol proposed by Norton et al. [11]; superior abdominal skinfold was taken along the midline between the inferior costal margin and the umbilicus; the posterior waist skinfold was taken along the posterior costal margin and demargin of the posterior axillary line; the trochanteric skinfold was measured 1 cm inferior to the gluteal fold in the lateral thigh area; the thigh skinfold was taken at 1 cm of the gluteal fold along the anterior midline on the inner thigh of the abductors area. Among the variables associated with the device, the type of handpiece used, the power, and the measured/indicated dose of the device were evaluated.

### 2.5. Procedure

Initially, a general analysis of the physical condition of each patient was made to identify the treatment areas and evaluate the type and state of the imperfection or pathology (localized adiposity, EFP, skin laxity) to define the suitability of the microwave treatment and the most adequate protocol. All patients were advised nutritionist support, and none exercised. Once the procedure was explained, a consent form was signed, and the previously described anthropometric parameters were measured in the treatment areas. These areas were delimited and then divided into secondary areas of 15 × 15 cm^2^. Skinfold test results by area were introduced in the microwave device that automatically selects the appropriate handpiece, which could be “Deep,” ideal for deep adiposity, or “Shallow” for cellulite, flaccidity, or adiposity with a fibrotic component, according to the degree given by the body caliper. The parameters of power and dosing pre-established by the device were used, adding 30% in each session with a 5°C cooling.

Skinfold areas with a thickness >2 cm (subdermal fat should be at least 1 cm thick) but <5.5 cm were identified. After cleaning with a normal saline solution, a vaseline layer was applied in the treatment area to obtain adequate contact between the handpiece and the skin, a better coupling, and greater fluidity of movement. The handpiece indicated by the device was used, keeping it perpendicular and in permanent contact with the skin, making soft continuous movements on the secondary zones for at least 7–10 minutes to cover the zone completely and homogeneously. The handpiece can be accessorised with contact and temperature sensors in order to assure a safe treatment. In order to carry out a lymphatic drainage, circular movements were performed within each demarcated treatment area, privileging a gradual ascending advancement along the lower limb and arm in the direction of the lymph node stations, repeating several steps until the exhaustion of the dose set for that area. The interval between sessions was 20–45 days following the previously described procedure.

### 2.6. Data Analysis

The participant's general characteristics description was made using absolute and relative frequencies for the qualitative variables. For quantitative variables, measures of central tendency and dispersion were used according to the data distribution determined by the Shapiro–Wilk test: for normal distribution, variables mean and SD (standard deviation), in non-normal median and interquartile range (IQR), were used. The data were summarized in tables. To establish localized adiposity improvement, a mean difference was proposed, using a repeated measures *t*-test for variables with normal distribution and the Wilcoxon signed-rank test for non-normal distributions, assuming a null hypothesis of a mean difference equal to 0. Results were verified with its respective confidence interval (CI) and *p* value (*p* values <0.05 were considered significant). Analysis of variance (ANOVA) using a repeated measures factor was used to ascertain whether the differences were statistically significant. A subgroup analysis was made for the variable BMI because of the possibility of a larger reduction with therapy in case of a higher body fat percentage (the overweight/obese group). To evaluate this, a mixed-design ANOVA was performed. Calculations were made using the Stata software version 16.

### 2.7. Biases

The presence of selection and measurement biases that could alter the results was considered. Selection bias could not be controlled, as for measurement bias, a single previously calibrated measuring instrument was used, and participants' physical evaluation was made by a sole investigator.

### 2.8. Ethical Aspects

The participant centers oversaw data confidentiality and informed consent by Colombian law (Ley 1581 de 2012). The procedures followed were in accordance with the Declaration of Helsinki. The authority provided approval and the corresponding ethical approval code.

## 3. Results

**Figure 2:**
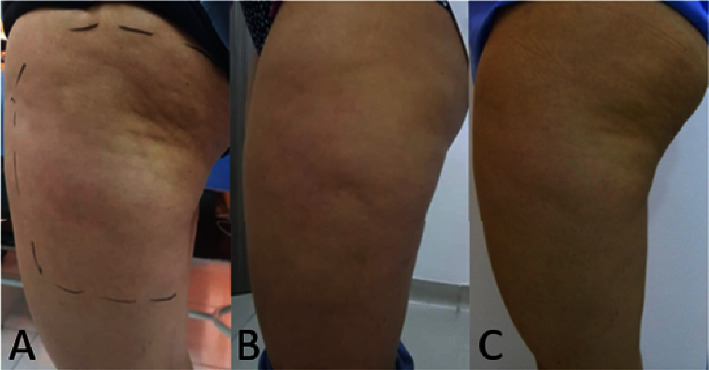
Photographic evaluation of patient's trochanteric skinfold before (A), 20 days after treatment (B), and 3 months after treatment (C).

**Figure 3:**
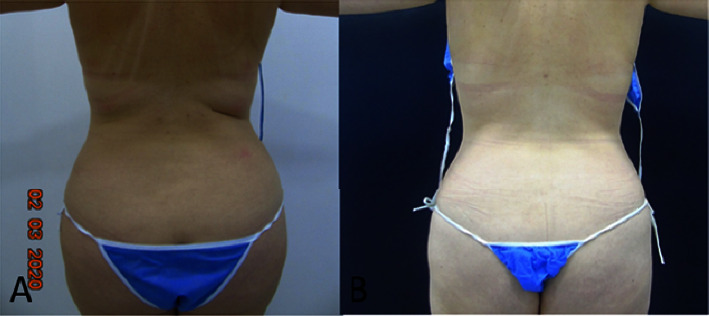
Photographic evaluation of patient's posterior waist before (A), and 3 months after treatment (B).

**Figure 4:**
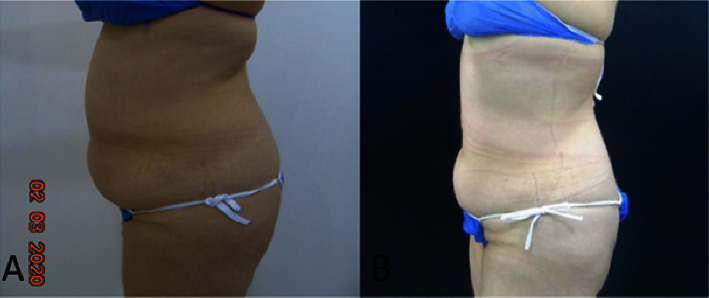
Photographic evaluation of patient's entire abdominal area before (A) and 3 months after treatment (B).

35 participants were included, 32 women (91.43%) and 3 men (8.57%); the mean age was 47.5 years (SD 9.00); 51.42% (*n* = 18) were married; the distribution among socioeconomic strata 3, 4, and 5 was similar; the highest educational level was a university in 91.43% (*n* = 32) of participants; 45.71% (*n* = 16) had a body mass index (BMI) of the overweight/obesity range previous to treatment; 85.71% (*n* = 30) had no past medical history of aesthetic surgery on the areas of interest; meanwhile, 14.49% (*n* = 5) have had liposuction, liposculpture, or abdominoplasty.  Table 1 describes the initial characteristics of the participants. All participants received three sessions of body remodeling using microwaves in the following areas: thigh 14.29% (*n* = 5), abdomen and back 34.29% (*n* = 12), in these three areas 40% (*n* = 14) and in the four areas (abdomen, back, thigh, and triceps) 11.43% (*n* = 4). Lower limbs were treated with a power range of 130–160 Watts and an energy dose range of 90–140 Jules using the “Shallow” handpiece in 63.2% of cases, and the trunk area was treated with a power range of 120–160 Watts and an Energy dose range of 80–150 Jules using the “Deep” handpiece in 61.4% of cases. Weight was *F*(1, 34) = 29.07 (*p* < 0.001), BMI was *F*(1, 34) = 27.42(*p* < 0.001), and skinfold test in all treatment areas resulted, specifically for abdominal perimeter *F*(1, 26) = 49.59 (*p* < 0.001), for superior abdomen fold *F*(1, 27) = 63.13; (*p* < 0.001), and for triceps *F*(1, 3) = 96.76 (*p*=0.002). Skinfold test showed a statistically significant reduction as reported in Table 2 and Figures 2–4.

### 3.1. Subgroup Analysis

Subgroup analysis was made for the BMI variable. Due to the low patient number with obesity, two groups were created, one group included those with a pretreatment BMI between 18.5 and 24.9 considered “normal” and another group with those who had a BMI equal or greater than 25 categorized as “overweight/obese”, and this was made to have similar sample sizes in each group. The mixed type variance analysis showed statistically significant differences between the category “BMI overweight/obese” compared with “normal” BMI in pretreatment weight (10.28; CI 95% 6.07–14.4 8), as well as post-treatment (7.77; CI 95% 3.57–11.97); in pre- and post-treatment abdominal perimeter (11.21; IC 95% 6.37–16.45 and 7.46; CI 95% 2.41–12.5), respectively; superior abdomen (12.97; CI 95% 7.69–18.25 and 9.56; CI 95% 4.28–14.85); subscapular skinfold (12.76; CI 95% 8.46–17.06 and 6.99; CI 95% 2.69–11.29); unlike what was found in abdominal (4.69; CI 95% −2.09–11.47 and 5.16; CI 95% −1.61–11.94), iliac crest (3.71; CI 95% −2.44–9.87 and 5.61; CI 95% −0.54–11.76), and trochanteric (3.62; CI 95% −9.97–17.22 and 1.13; CI 95% −12.46–14.73) skinfolds, where no differences were found. Posterior waist skinfold showed differences in pretreatment BMI but not in post-treatment between the two groups (7.53; CI 95% 0.94–14.13 and 5.15; CI 95% −1.45–11.74). In the intragroup analysis, when comparing both moments, no statistically significant differences were found in weight, both in the normal BMI group and the overweight/obese group (−1.74; CI 95% −5.76–2.28 and −4.24; IC 95% −8.62–0.14), nor in the trochanteric fold (−9.81; CI 95% −19.94–0.33 and −12.3; CI 95% −28.64–4.04). Differences were found in the abdominal perimeter (−7.12; CI 95% −12.25–−1.98 and −11.07; CI 95% −16.02–−6.12), in the normal BMI group, and in the overweight/obese group, respectively, as well as in the abdominal skinfold (−9.79; CI 95% −16.44–−3.13 and −9.31; CI 95% −16.21–−2.40), iliac crest (−11.59; CI 95% −17.99–−5.19 and −9.69; CI 95% −15.58–−3.80), subscapular (−4.82; CI 95% −9.12–−0.42 and −10.58; CI 95% −14.79–−6.38); meanwhile, in the superior abdominal skinfold, differences were found in the overweight/obese group but not in the normal BMI (−8.87; CI 95% −13.96–−3.78 and −5.46; CI 95% −10.93–0.01), respectively, as well as in the posterior waist (−9.09; CI 95% −15.53–−2.65 and −6.7; CI95% −13.45–0.05).

## 4. Discussion

Because 80% of the microwave energy is focused on the adipocytes and the remaining 20% is absorbed by the epidermal and dermal layers, hence, there is no risk of causing overheating points on the skin and the energy achieves a greater depth [12]. Differences are found in studies concerning the effectiveness of the microwave system in the treatment of localized adiposity [1–4, 13]; this study is the first in a Latin-American population, in which a reduction in the skinfold test measurements of different body zones is achieved using this system (2.45 GHz). All patients were Colombian, with a strong female predominance as reported in the literature [1, 14] that could be associated with the anatomical, genetic, and hormonal predisposition for developing alterations in the adipose tissue when comparing them with men [6], as well as the greater propensity for aesthetics in females. The average age was 47 years, and most patients had a university education (91.34%), which could indicate that a higher educational level has a greater tendency for self-care and a better economic income, allowing access to this service and a greater disposition to improve their quality of life. The 14.49% of patients had a past medical history of an aesthetic surgical intervention, and despite this being the gold standard for treating localized fat, it does not act on skin laxity or cellulite; additionally, the current tendency is toward nonsurgical procedures, more conservative and with less adverse effects. Different parameters were applied according to the skinfold test results and the visual physical exam, with individualized treatment for each patient. Although the device has predetermined parameters for the handpiece selection, a standardized parameter cannot be given without proper measuring and evaluation of each patient. The “Shallow” handpiece was used for the treatment of EFP, improving and dissolving fibrosis as well as enhancing skin tension, while, in the abdomen, the “Deep” handpiece was used to favor adipolysis of localized fat. Both handpieces were used in different treatment sessions. The predominant fat distribution was abdominal and in the trochanteric area. In abdominal fat, one of the most important measurements when treating weight loss is the abdominal perimeter is a cardiovascular and metabolic syndrome predictive factor, independent of the patient's BMI [10]. After three sessions of microwave, differences were found in pre and post-treatment abdominal perimeter with a median of 7 cm (*z* = 4.545, *p* < 0.001), showing a significant reduction in abdominal adiposity greater than that reported by Bonan et al. [4], where a series of 12 patients between 29 and 55 years were subjected to 4 sessions during a month using the microwave system achieving a mean reduction of 3.90 cm (range 7–15 cm) in abdominal adiposity, also surpassing the results by Nisticó et al. [5], which showed a mean decrease of 4.2 cm (*p* < 0,001) in abdominal circumference, and corroborating the use of noninvasive technologies such as microwaves as promising for the treatment of abdominal adiposity. Until now, the effect of the microwave system on the relationship between obesity and cardiovascular risk is not known because it does not impact visceral fat. Indeed, the Onda system represents a noninvasive device that acts on the subdermal fat layer. The superior abdominal skinfolds decrease by 8.8 mm in the overweight/obese group (*p*=0.001), while the normal BMI group showed a tendency towards the reduction with a value of 5.46 mm (*p*=0.001); in the inferior abdomen, both in subjects with a normal BMI and overweight/obese, the reduction was similar (9.79 mm, *p*=0.001 and 9.31 mm, *p*=0.001, respectively). Interestingly, in patients with a BMI above 25, the changes in variables (abdominal perimeter, superior abdominal fold, subscapular fold, posterior waist) were greater than those with a normal BMI, and this could be due to a wider field of action for massive adipolysis. 54.29% of patients had an initial normal BMI while 45.71% were in the range of overweight/obese, which in the context, the Colombian population is slightly lower than the prevalence of overweight and obesity in adults (57.5%) [10]. Weight reduction pre and post-treatment were observed in all patients with a median of 2.3 kg (*z* = 4.684, *p* < 0.001), and despite no intervention in physical activity or nutritional aspects, these findings were not evidenced in previous studies in which no statically significant differences were found in BMI as reported by Di Pietro et al. [1], Bennardo et al. [3], and Bonan et al. [4]. Weight loss (generalized adiposity) was an additional effect, but not the general objective of this study, it is necessary to determine the body composition with systems such as bioelectrical impedance to determine the fat percentage, lean mass, and total body water to better characterize where these changes occur, which is why future investigators are advised to use it in all their patients. Regarding the adiposities of the trochanteric and thigh regions, a decrease in measurements between the beginning and end of treatment was observed with a mean decrease in the inner thigh of 9.73 mm (CI 95% 6.78–12.68) and the external aspect with a median decrease of 7 mm (*z* = 3.825, *p*=0.001), the reduction of localized adiposity in the folds of the iliac crests was the variable with the greatest difference before and after the procedure with a decrease of 11.59 mm (CI 95% 7.68–15.50) in subjects with normal BMI and 9.69 mm (CI 95% 7.36–12.02) in patients with overweight/obesity, such that its use for these areas is highly recommended. These results are not comparable with other studies since we used skinfolds and other studies used perimeter or circumference; Bonan et al. [4] showed a decrease of 2.8 cm (range 2.5–3 cm) in trochanteric adiposity, as well as the study conducted by Bennardo et al. in 2022 [3] who reported a decrease in the gluteal circumference of 4.3 cm and thighs of 2.1 cm. These differences could be related to the ethnic characteristics of body constitution in the Latin population, where a major fat distribution is observed in these areas that could explain the lesser loss. With regard to the cellulite appearance, different scales are found, as reported by Di Pietro et al. [1], and in this study, they described a series of 20 women, with a BMI <30, who underwent four sessions of microwave treatment with a 30-day interval; they later compared results before the first session, four and eight weeks after treatment using the cellulite severity scale-CSS [14]. An initial moderate to severe CSS was found in 95% of patients with posterior significant improvement in cellulite degree, and 80% of patients were reclassified as mild (*p* < 0.05), with no loose skin in 65% of patients. Although, in the present study, a visual or satisfaction scale was not considered, the procedure and integral management of patient perception were satisfactory, with many patients booking additional sessions after their three initial sessions due to a good response to treatment (Figures 2–4). Among the reported adverse effects, mild erythema was noted as described by Bennardo et al. [3] as well as a slight burning sensation lasting two minutes after the end of the procedure. Symptoms such as pruritus, numbness, rigidity, inflammation, burns, nodules, and blistering on the treatment zone were not observed in any case with no serious adverse effects reported, as described by Di Pietroet al. [1] or Bonanet al. [4]. The combination of microwaves and other therapies to enhance results has gained interest in recent publications as described by Nisticò et al. [5], where the microwave system and magnetic stimulation showed a reduction in waist circumference and improvement in skin laxity in all patients (20 women and 5 men) (*p* < 0.001), and this should be considered in future studies with combined technologies.

The Shapiro–Wilk test is a statistical hypothesis test that is used to determine whether or not a given data set is normally distributed [15].

The Shapiro–Wilk test and the Kolmogorov–Smirnov test are two well-known and historically commonly employed quantitative methods for determining data normality [16].

Both tests compare the study sample's scores to a normally distributed set of scores with the same mean and standard deviation; their null hypothesis is that the sample distribution is normal.

The Shapiro–Wilk test was selected for this study since it is more appropriate for small sample sizes (*N* ≤ 50), but it can also be validly applied to large sample sizes. Furthermore, the Shapiro–Wilk test provides greater power than the Kolmogorov–Smirnov test. For these reasons, the Shapiro–Wilk test has been recommended as the numerical means for assessing data normality [17, 18]. In addition, Wilcoxon signed-rank test (WSR) was used in this research for non-normal distributions, assuming a null hypothesis of a mean difference equal to 0.

The WSR was more often the more powerful test, and the magnitude of the WSR's power advantage often increased with sample size [19]; it has salient advantages over the one-sample *t*-test for testing the null hypothesis [20]. The main benefit of the Wilcoxon signed-rank test is that it does not depend on the parameters or the form of the parent distribution. No assumptions regarding the distribution's shape are necessary. For this reason, this test is often used as an alternative to *t*-tests whenever the population cannot be assumed to be normally distributed [21].

Study limitations included a lack of a control group, a small sample size, similar to that found in the literature, a small number of men included, which is not enough to establish comparisons, and given the differences between android and gynoid morphology, it is not possible to extrapolate female results to males. Despite this, male subjects had a successful fat decrease in localized fat. Future studies should delve into this to establish sex differences.

## 5. Conclusions

This is the first study using the microwave system in a Latin-American population for body contour treatment. The positive results particularly achieved by the skinfold test in all treatment areas, which showed a statistically significant reduction of localized adiposity, and the lack of major adverse effects makes this technique an excellent option for the treatment of patients seeking body remodeling. Continued research in this area with methods that allow a more objective measurement of body fat, studies with a larger sample, inclusion of a male population, and evaluation of long-term outcomes (> six months) are strongly encouraged to improve the current evidence.

## Figures and Tables

**Table 1:** Sociodemographic characteristics and medical history.

Sociodemographic characteristics and medical history	*n* = 35 (%)
*Sex*	
Women	32 (91.43)
Men	3 (8.57)

*Marital status*	
Married	18 (51.42)
Single	13 (37.14)
Divorced	2 (5.72)
Cohabitants	1 (2.86)
No data	1 (2.86)

*Socioeconomic levels*	
3	10 (28.57)
4	12 (34.29)
5	11 (31.43)
6	2 (5.71)

*Educational level*	
Secondary school	2 (5.71)
Bachelor's degree	32 (91.34)
Postgraduate	1 (2.86)

*Labor*	
Health	6 (17.14)
Lawyer	3 (8.57)
Finance/economics	4 (11.43)
Commerce	7 (20)
Arts	2 (5.71)
Engineering	2 (5.71)
Others^*∗*^	11 (31.43)

*Body mass index (BMI)*	
Normal	19 (54.29)
Overweight	12 (34.28)
Obese	4 (11.43)

*Previous aesthetic surgery*	
Yes	5 (14.29)
No	30 (85.71)

*Menopause (n* *=* *32)*	
Yes	8 (25)
No	24 (75)

*Current use of contraceptives (n* *=* *32)*	
Yes	3 (9.38)
No	29 (90.63)

^*∗*^Others include employed, self-employed, teacher, social communicator.

**Table 2:** Pre and post-treatment measurements.

Variables	Pretreatment	Post-treatment	*F* (df)	*p*
	Mean (SD)	Mean (SD)		
Weight	66.58 (8.26)	63.69 (6.99)	29.07 (1, 34)	0.001
BMI	25.24 (3.42)	24.14 (2.67)	27.42 (1, 34)	0.001
Waist perimeter	92.15 (9.15)	82.98 (6.79)	49.59 (1, 26)	0.001
Superior abdomen skinfold	27.18 (10.09)	19.89 (7.63)	63.13 (1, 27)	0.001
Abdominal skinfold	31.19 (9.92)	21.63 (7.87)	82.32 (1, 26)	0.001
Iliac crest skinfold	37.38 (8.20)	26.81 (7.12)	114.33 (1, 23)	0.001
Subscapular skinfold	29.57 (8.63)	21.74 (5.53)	67.47 (1, 22)	0.001
Posterior waist skinfold	34.05 (8.22)	26.10 (7.74)	27.36 (1, 20)	0.001
Trochanteric skinfold	46.08 (12.52)	35.58 (12.21)	26.94 (1, 17)	0.001
Inner thigh skinfold	38.8 (9.64)	29.07 (8.10)	50.18 (1, 14)	0.001
Tricipital skinfold	26.38 (3.45)	18.25 (2.06)	96.76 (1, 3)	0.002

## Data Availability

The data supporting the current study are available from the corresponding author upon request.
